# MRgFUS treatment of superficial osteoid osteomas of the lower limbs

**DOI:** 10.1186/2050-5736-3-S1-O45

**Published:** 2015-06-30

**Authors:** Alberto Bazzocchi, Alessandro Napoli, Giacomo Filonzi, Giancarlo Facchini, Paolo Spinnato, Maurizio Busacca, Carlo Catalano, Ugo Albisinni

**Affiliations:** 1 The “Rizzoli” Orthopaedic Institute, Bologna, Italy; 2 University of Rome – Sapienza, Rome, Italy

## Background/introduction

Osteoid osteoma (OO) is a relatively common benign bone tumor (2-3% of all bone tumors, 10% of benign bone tumors) usually developing in children and young adults. It is quite painful and patients typically complain of localized pain that is worse at night and characteristically relieved by non-steroid anti-inflammatory drugs (NSAIDs). Conventional treatment options include surgery, systemic drugs administration, and imaging guided percutaneous procedures. Minimally invasive procedures are considered of primary importance, and this is even enhanced considering the benign nature of the lesion and the young age of the affected population. CT guided radiofrequency ablation (CTgRFA) is the most popular percutaneous technique with clinical success rate reported between 85% and 98%. However, CTgRFA is still rather invasive, and it requires CT-guidance, with obvious concerns linked to radiosensitivity especially of young patients. MR guided focused ultrasound surgery (MRgFUS) is a minimally invasive procedure that can be performed relatively fast, in a single session and with limited amount of energy deposition and no use of ionizing radiation.

## Methods

From March 2013 to May 2014, 7 consecutive patients (6M, 1F; mean age 33.5±12.4, range 19-64 years old) with superficial osteoid osteomas of the lower limb were treated at our Institute with MRgFUS (ExAblate 2100, InSightec). Six lesions were located at the femur, one at the tibia. The mean time between the onset of symptoms and the diagnosis was 3.75 months (range 1-9 months). One patient had previously undergone CTgRFA (in a different hospital) but experienced a relapse of symptoms 4 months after, and imaging revealed that the lesion was still there. For all other patients MRgFUS was used as first-line treatment (apart from NSAIDs). Patients were examined clinically (visual analogue scale score – VAS; Qol) at baseline and at 1, 3, 6 and 12 months of follow-up.

## Results and conclusions

In five patient the 12-month follow-up period has been completed, while two patients are still at 1- and 3-month check points. The mean VAS at the baseline was 7.5. In all patients VAS dropped to 0 after 1 month. In 6 patients (86%) VAS remained 0 during the follow-up, while in 1 patient VAS dropped from 9 to 0 after 1 month but rose to 2 after 3 months (6-month control available, no recurrence documented). No intra-operative complications or short/mid-terms adverse events were observed.

These preliminary data showed that MRgFUS can be effectively adopted for the treatment of superficial OO and can be performed safely and with a high rate of success for the noninvasive treatment of this condition.

